# Multi-omics reveals new links between Fructosamine-3-Kinase (FN3K) and core metabolic pathways

**DOI:** 10.1038/s41540-024-00390-0

**Published:** 2024-06-03

**Authors:** Safal Shrestha, Rahil Taujale, Samiksha Katiyar, Natarajan Kannan

**Affiliations:** 1 Institute of Bioinformatics, University of Georgia, Athens, GA USA; 2 Department of Biochemistry and Molecular Biology, University of Georgia, Athens, GA USA

**Keywords:** Systems biology, Biochemistry

## Abstract

Fructosamine-3-kinases (FN3Ks) are a conserved family of repair enzymes that phosphorylate reactive sugars attached to lysine residues in peptides and proteins. Although FN3Ks are present across the Tree of Life and share detectable sequence similarity to eukaryotic protein kinases, the biological processes regulated by these kinases are largely unknown. To address this knowledge gap, we leveraged the FN3K CRISPR Knock-Out (KO) HepG2 cell line alongside an integrative multi-omics study combining transcriptomics, metabolomics, and interactomics to place these enzymes in a pathway context. The integrative analyses revealed the enrichment of pathways related to oxidative stress response, lipid biosynthesis (cholesterol and fatty acids), and carbon and co-factor metabolism. Moreover, enrichment of nicotinamide adenine dinucleotide (NAD) binding proteins and localization of human FN3K (HsFN3K) to mitochondria suggests potential links between FN3K and NAD-mediated energy metabolism and redox balance. We report specific binding of HsFN3K to NAD compounds in a metal and concentration-dependent manner and provide insight into their binding mode using modeling and experimental site-directed mutagenesis. Our studies provide a framework for targeting these understudied kinases in diabetic complications and metabolic disorders where redox balance and NAD-dependent metabolic processes are altered.

## Introduction

Reducing sugars including ribose and glucose can spontaneously react with free amines on peptides, proteins, and lipids through a process called glycation^1^. This process can occur either endogenously or exogenously via the foods we consume^2^. Because increased glycation can negatively impact biomolecular functions through the formation of stable adducts, known as ketosamines^3^, organisms have evolved enzymatic repair systems as a defense mechanism. Fructosamine-3 kinases (FN3Ks) are one such family of repair enzymes that use Adenosine triphosphate (ATP) to de-glycate lysine residues in proteins^4–6^. They do so by catalyzing the transfer of the gamma phosphate from ATP to the 3’ hydroxyl group in the ketosamine substrate, which rapidly breaks down into inorganic phosphate and 3-deoxyglucosone (3-DG), regenerating the original lysine^6^. There is emerging evidence that altered FN3K expression and increased ketosamine levels contribute to metabolic disorders^7,8^ through the production of Advanced Glycation End (AGEs) products^9^ and reactive oxygen species (ROS)^10^.

Deglycation by FN3Ks is an ancient mechanism for protein repair as FN3K homologs are found throughout the tree of life^11,12^. While lower eukaryotes and prokaryotes possess a single copy of the FN3K gene, most tetrapod genomes contain two: FN3K and FN3K Related Protein (FN3KRP). Interestingly, two independent gene duplication events have led to the tetrapods having two copies: one in reptiles/birds and the other in placental mammals^13^. It is believed that in lower organisms, FN3K orthologs repair proteins glycated by ribose-5-phosphate, a potent glycating metabolite formed from the conserved pentose phosphate pathway^11,12^. Despite the remarkable conservation of FN3Ks across diverse organisms and their emerging role in protein repair, the biological and cellular processes linked to this important class of proteins remain unknown.

**Fig. 1:**
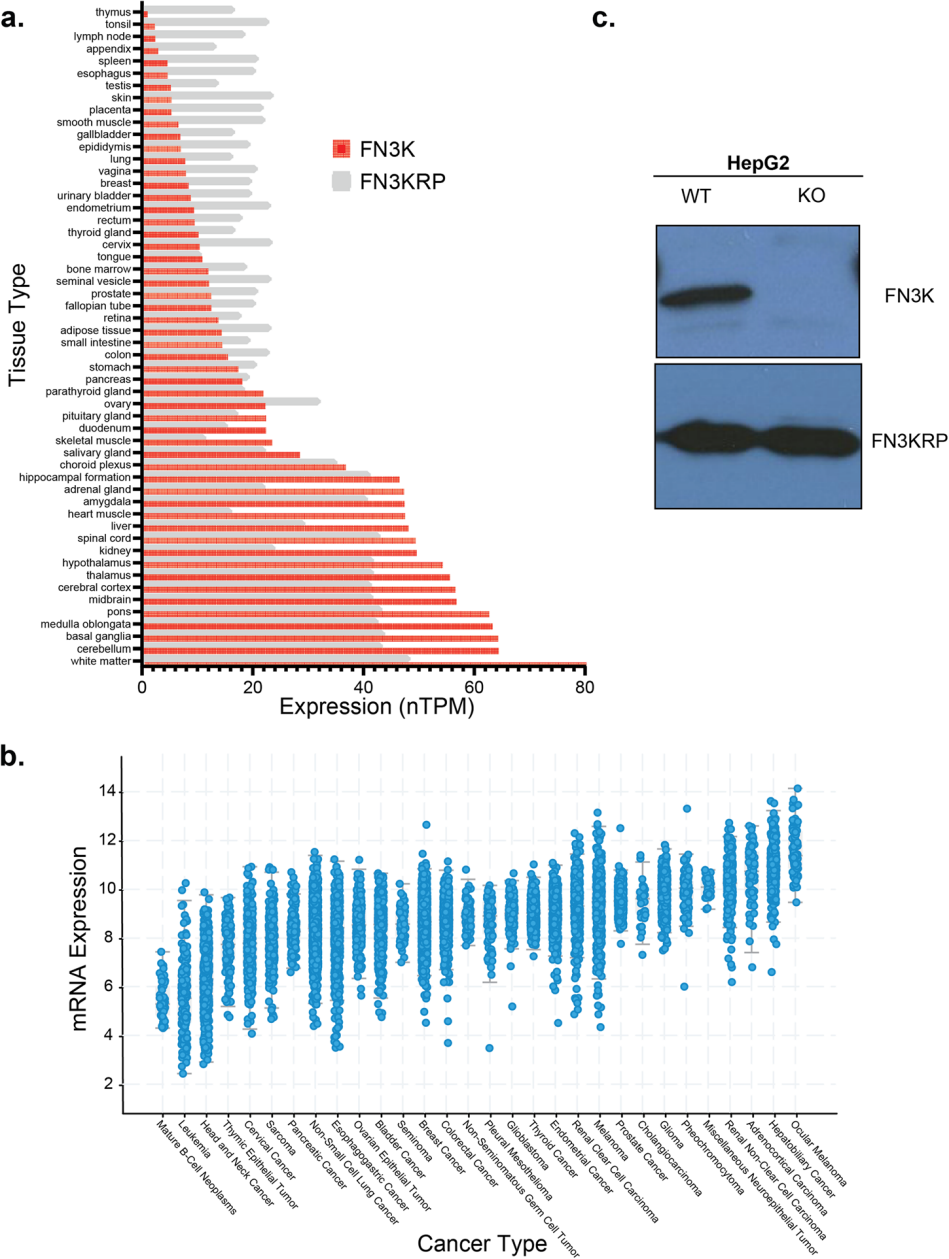
Study of FN3K expression in tissues and cancer cells. **a** Tissue-specific expression levels of human FN3K (HsFN3K) and human FN3KRP (HsFN3KRP), based on data from the Protein Atlas Database. **b** The levels of HsFN3K RNA expression across various types of cancer, arranged by median expression values. The figure was produced using cBioPortal^57^. Unit for mRNA expression levels: RSEM (Batch normalized from Illumina HiSeq_RNASeqV2) (log2(value + 1)). **c** Western blot analysis displaying the total quantities of HsFN3K and HsFN3KRP in both wild-type and FN3K knockout (KO) HepG2 cells. The blots were from separate gels.

FN3K activity is highly substrate-specific with human FN3K (HsFN3K) phosphorylating ketosamines resulting from glycation of both L and D orientation sugars, whereas FN3KRP orthologs are limited to only D-orientation sugars^11,12,14^. At the transcriptional level, in human tissues, HsFN3K is highly expressed in the brain, kidney, liver, heart muscle and adrenal gland (Fig. 1a) whereas HsFN3KRP expression levels are somewhat uniform throughout the different tissues (Fig. 1a). The paralogs are also reported to be localized to distinct subcellular compartments. Based on immunohistochemistry (IHC) studies on HsFN3K in HepG2 cells, HsFN3K is reported to be localized in mitochondria whereas HsFN3KRP is localized in nucleoplasm in different cell lines^15^.

Previously, we reported the crystal structure of FN3K ortholog from *Arabidopsis thaliana* (AtFN3K), revealing an evolutionarily conserved redox regulation mechanism involving conserved cysteines in the ATP binding P-loop^16^. Specifically, we demonstrated that AtFN3K adopts a disulfide-linked dimer that can be reversed under reducing conditions and that the equivalent cysteine in HsFN3K (C24^HsFN3K^) performs an analogous role conferring redox sensitivity and disulfide-mediated oligomerization. Furthermore, a recent study has proposed a potential link between HsFN3K deglycation function and the development of hepatocellular carcinoma (liver cancer) through the involvement of the nuclear transcription factor Nuclear factor erythroid 2-related factor 2 (NRF2)^17^, which controls the expression of multiple antioxidant enzymes^18^. Acting as an adaptor protein, Kelch-like ECH-associated protein (KEAP1) interacts and directly inhibits Nuclear factor erythroid 2-related factor 2 (NRF2) by facilitating its ubiquitination through Cullin3 (CUL3) and marking it for proteasomal degradation^19^. KEAP1 has redox-sensitive cysteines that undergo modifications during oxidative stress, impeding the interaction between KEAP1 and NRF2. The progressive buildup of NRF2 triggers the induction of antioxidant enzymes, further underscoring the role of HsFN3K in maintaining cellular redox balance and altered expression in liver cancer (Fig. 1b). It is worth noting that higher HsFN3K expression levels are also observed in ocular/eye and adrenal cancers (Fig. 1b). However, despite the altered expression of HsFN3K in multiple cancer types and their established roles in metabolic diseases such as diabetes and associated complications^20,21^, our understanding of FN3K’s functions within human cells and across different species, particularly in the context of redox regulation, remains incomplete.

Here we leverage the CRISPR Knock Out (KO) of HsFN3K in HepG2 liver cancer cell line (Fig. 1c, Supplementary Fig. 1) and employ a multi-omics approach integrating transcriptomics, metabolomics, and interactomics to illuminate the cellular pathways associated with HsFN3K. Notably, knocking out HsFN3K did not change protein levels for the paralog HsFN3KRP (Fig. 1c). Completely knocking out HsFN3K in HepG2 cells resulted in upregulation of lipid biosynthesis (fatty acid and cholesterol) and oxidative stress response pathways. Joint enrichment analysis of the transcriptomics and the metabolomics datasets revealed enrichment of pathways related to glutathione and co-factor metabolisms, including Coenzyme A (CoA) and nicotinate/nicotinamide metabolism. Interestingly, several of the HsFN3K interacting partners were involved in fatty acid and pyruvate metabolism pathways, which we further validated using immunoprecipitation in HepG2 cells. We found that HsFN3K interacts with Fatty acid synthase (FASN) and Lactate dehydrogenase A (LDHA) in cytoplasm. Moreover, by performing enrichment analysis on the integrative network combining transcriptomics and interactomics, we identified enrichment of Nicotinamide dinucleotide (NAD) binding superfamily and thiolase domains. Finally, we validated HsFN3K binding to NAD compounds using Differential Scanning Fluorimetry (DSF). We further probed the binding specificity through mutagenesis and identified key residues in the adenosine as well as substrate binding pockets, revealing that HsFN3K is inhibited by NADH.

## Results

**Fig. 2:**
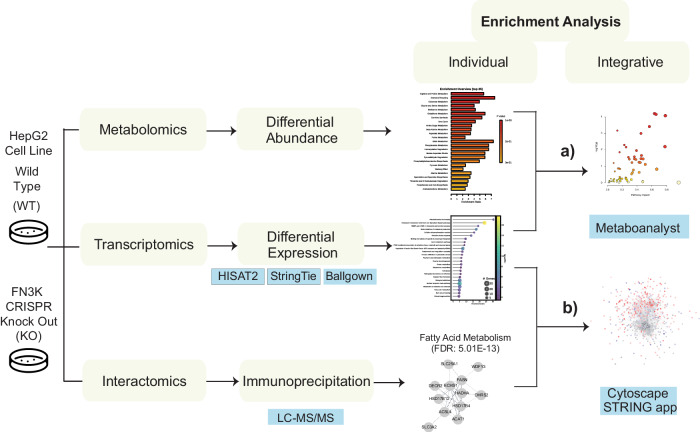
Schematic diagram describing the integrative analyses on the multi-omics datasets. FN3K CRISPR KO HepG2 liver cancer cell lines were leveraged to perform multi-omics analyses: transcriptomics, metabolomics and interactomics. Metaboanalyst and network based Cytoscape framework were used to perform the integrative analyses.

To investigate the molecular pathways regulated by HsFN3K, we utilized an integrative multi-omics approach. By leveraging the FN3K Clustered Regularly Interspaced Short Palindromic Repeats (CRISPR) Knock-Out (KO) HepG2 cell lines, we identified genes, metabolites, and pathways altered by HsFN3K knock out. Enrichment analyses on individual datasets as well as combined datasets (metabolomics and transcriptomics) (Fig. 2a, b) highlighted key pathways (Supplementary Fig. 2a) placing these enzymes in a biological context.

### Transcriptomic profiling and pathway analysis of HsFN3K KO cells reveal alterations in metallothionein binding, lipid metabolism, and oxidative stress response pathways

**Fig. 3:**
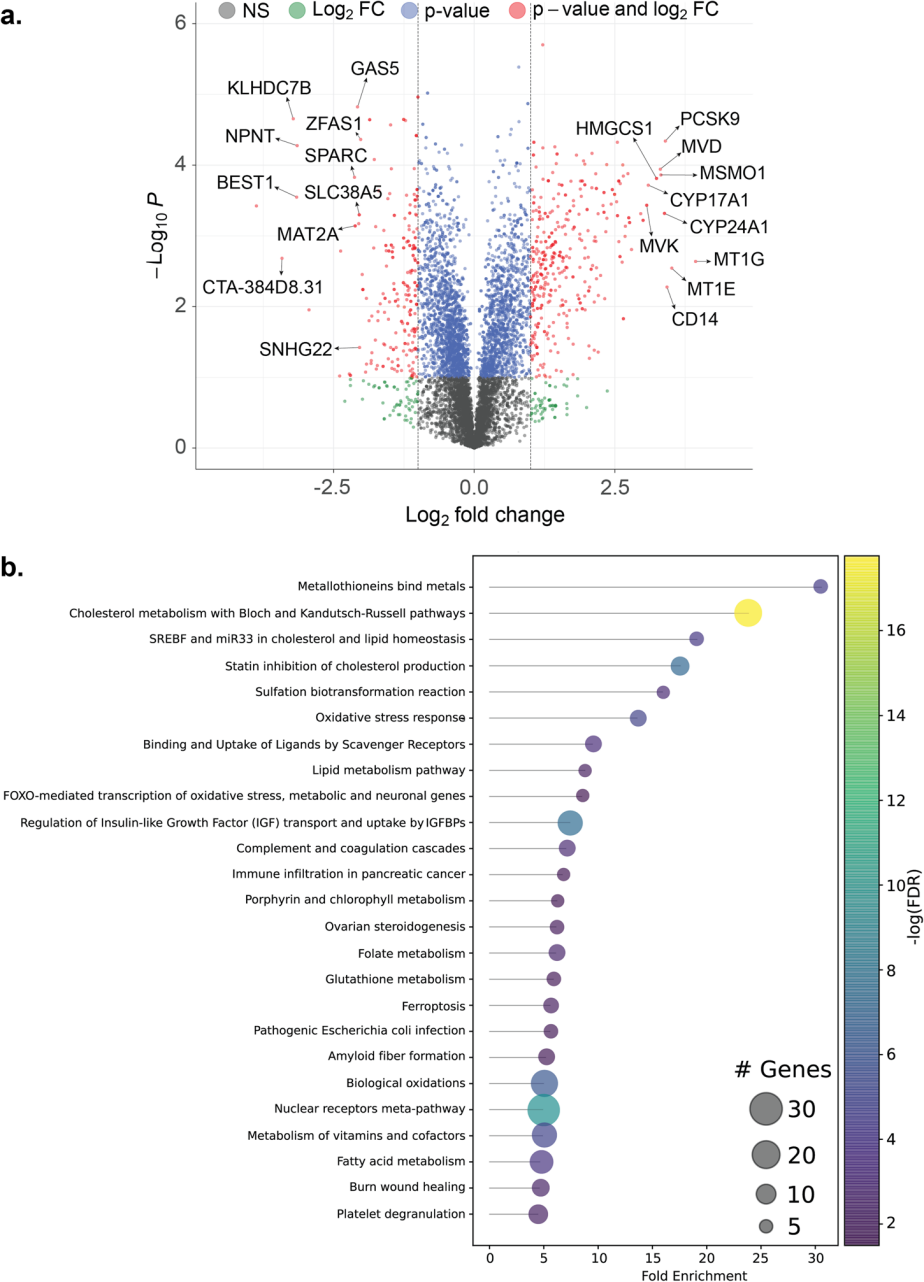
Identification of differentially expressed genes (DEG). **a** A volcano plot exhibiting significantly upregulated and downregulated genes in the FN3K knockout (KO) HepG2 cell line compared to the wild type HepG2 cell line. **b** A lollipop graph showcasing enriched biological pathways based on DEGs as ordered according to fold enrichment.

For the transcriptomics, we conducted RNA sequencing on total RNA from the KO and the wild type (WT) cells. The obtained reads were analyzed using the HISAT2-StringTie-Ballgown^22^ suite of tools where the reads are first mapped against the human reference using HISAT2^23^, followed by transcript assembly and quantification using StringTie^24^. Finally, based on these quantified transcripts in both the KO and WT cell lines, Ballgown^25^ was used to perform a differential expression analysis. Overall, we identified 408 differentially expressed genes (DEGs) (greater than 2 log-fold change and p-value < 0.05) in the CRISPR KO cells relative to WT cells) (Fig. 3a). Out of the 408 genes, 305 genes were upregulated. The DEGs also include 23 genes that are RNA genes or pseudogenes (Supplementary Dataset File 1).

The most upregulated genes include metallothioneins (MT) paralogs such as MT1E (11-fold) and MT1G (15-fold) and members of the Cytochrome P450 family such as CYP24A1 (10-fold) and CYP17A1 (8.5-fold) (Fig. 3a). Moreover, genes in cholesterol synthesis and homeostasis: Proprotein convertase subtilisin/kexin type 9 (PCSK9) (10.5-fold), methylsterol monooxygenase 1 (MSMO1) (10-fold), mevalonate diphosphate decarboxylase (MVD) (10-fold), mevalonate kinase (MVK) (8-fold), and 3-hydroxy-3-methylglutaryl-CoA synthase 1 (HMGCS1) (9.5-fold) were also highly upregulated (Fig. 3a).

The most downregulated genes include the kelch domain containing 7B (KLHDC7B) (9.2-fold) and its long noncoding RNA counterpart CTA-384D8.31 (10.7-fold) (Fig. 3a). Other RNA genes such as growth arrest specific 5 (GAS5) (4.2-fold) and small nucleolar RNA host gene 22 (SNHG22) (4.1-fold) were also downregulated (Fig. 3a). Moreover, there was down regulation of genes involved in cell growth secreted protein acidic and cysteine rich (SPARC) (4.3-fold) and in cell adhesion nephronectin (NPNT) (8.9-fold) in the KO (Fig. 3a). Interestingly, S-adenosylmethionine (SAM) synthesizing gene methionine adenosyltransferase 2 A (MAT2A) (4.3-fold) was among the top 10 most down-regulated genes (Fig. 3a).

After identifying differentially expressed genes, we performed enrichment analysis at the pathway level, prioritizing them according to their fold enrichment. This approach enabled us to effectively quantify the overrepresentation of specific pathways by comparing the total number of genes identified in each pathway against the background frequency of genes annotated for that pathway^26^. Notably, the pathway involving “Metallothioneins bind metals” emerged as the most significantly enriched pathway, as determined by fold enrichment (Fig. 3b). Metallothioneins are low molecular weight (6–7 kDa) cysteine-rich proteins that play crucial roles in metal homeostasis and the oxidative stress response pathway, providing protection against DNA damage and cytotoxicity^27^.

Following this, we observed significant enrichment in pathways associated with lipid biosynthesis and signaling, particularly in cholesterol and fatty acid metabolism (Fig. 3b). Other notable pathways were related to “Oxidative stress response” and related pathways such as “Binding and Uptake of Ligands by Scavenger Receptors”, “FOXO-mediated transcription of oxidative stress, metabolic, and neuronal genes”, “Glutathione metabolism” as well as “Ferroptosis”. Finally, “Vitamins and co-factor metabolism” as well as related pathways such as “Folate metabolism” and “Porphyrin and chlorophyll metabolism” were also enriched (Fig. 3b).

### Joint pathway analysis using transcriptomics and metabolomics datasets reveal enrichment of glutathione, carbon, and co-factor metabolisms including nicotinate/nicotinamide metabolism

We previously identified differentially abundant metabolites by comparing the metabolic profiles of FN3K KO and WT HepG2 cells^16^. Metabolite set enrichment analysis (MSEA) of the differentially abundant metabolites revealed an enrichment in several pathways related to amino acid metabolism, in addition to pathways such as “Glutathione metabolism”, “Pyruvate metabolism”, and “Pantothenate and CoA Biosynthesis” (Supplementary Fig. 2b). Integrating this with our transcriptomics data, we conducted a joint pathway analysis (Fig. 2a) using MetaboAnalyst^28^ to understand the interplay between the two datasets. Furthermore, we employed the STITCH plugin in Cytoscape to visualize the interactions between differentially expressed genes and differentially abundant metabolites. Details with matched features for the top enriched pathways are provided in Supplementary Dataset File 1.

Matched features for enriched pathways such as “Terpenoid backbone biosynthesis”, “Retinol metabolism”, and “Steroid biosynthesis” were exclusive to differentially expressed genes. In contrast, “Aminoacyl-tRNA biosynthesis” and “Valine, leucine, and isoleucine biosynthesis” were exclusive to differentially abundant metabolites. However, several of the enriched pathways matched both datasets. The highly enriched “Glutathione metabolism” pathway included metabolites such as glutathione, glycine, and glutamate as well as genes such as glutathione peroxidase 3 (GPX3), glucose-6-phosphate dehydrogenase (G6PD), N-acetyltransferase 8 (NAT8), glutathione S-transferase alpha 1 (GSTA1), microsomal glutathione S-transferase 1 (MGST1), and Ribonucleotide reductase regulatory subunit M2 (RRM2) (Supplementary Dataset File 1).

**Fig. 4:**
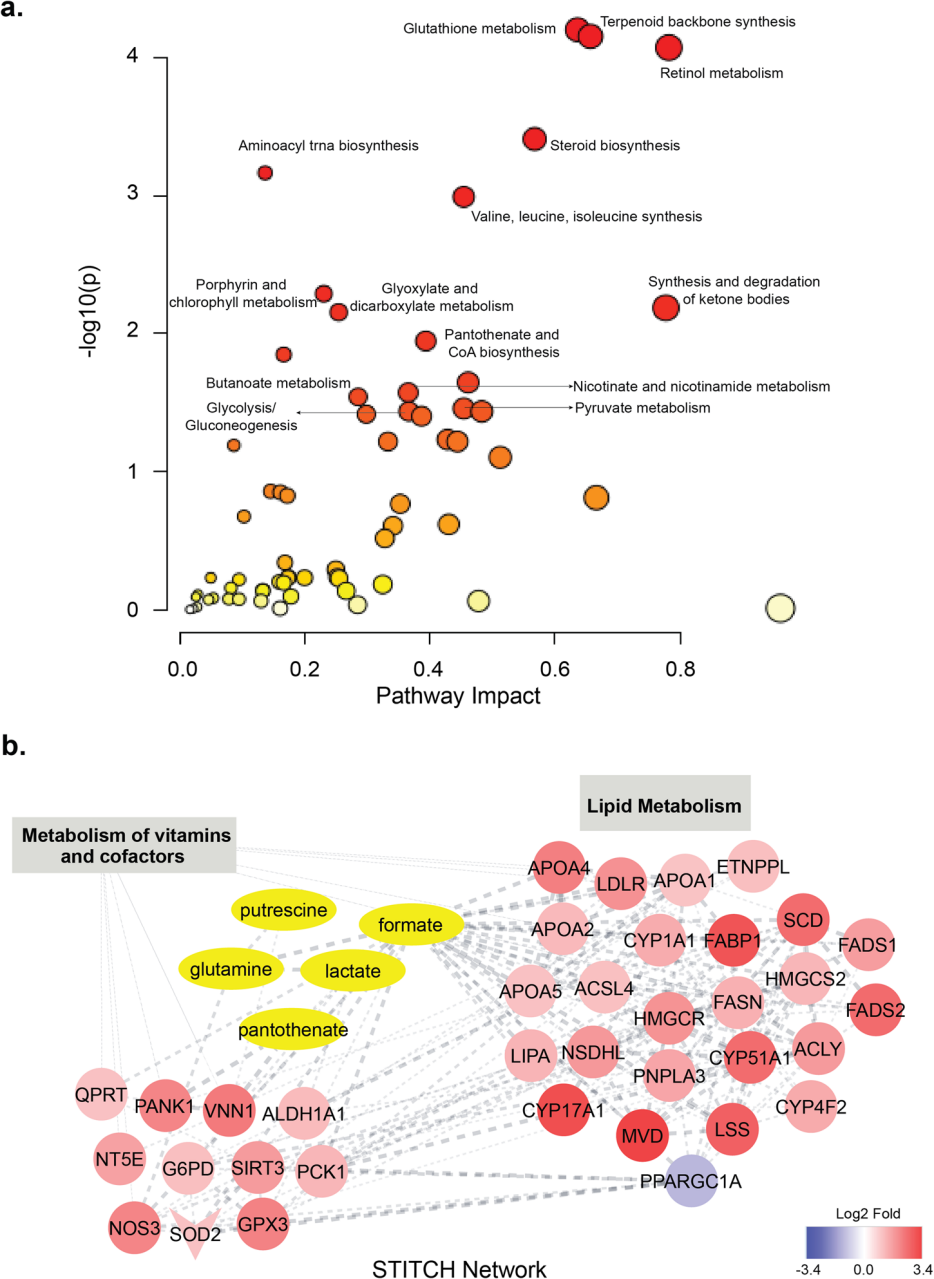
Joint pathway analysis of the transcriptomics and metabolomics datasets. **a** Plot showing enrichment of various pathways associated with glutathione, carbon, and co-factor metabolisms as identified through the Metaboanalyst tool. **b** STITCH network showing associations between DEGs and DAMs in the context of vitamin and co-factor metabolisms.

Moreover, we identified key pathways enriched in the context of carbon and co-factor metabolisms. Specifically, pathways related to carbon metabolism such as “Glycolysis/Gluconeogenesis”, and “Pyruvate Metabolism” was enriched (Fig. 4a) and MSEA identified potential links between these metabolic pathways and Glyoxalase I (GLO1) gene expression (Supplementary Dataset File 1). Similarly, the metabolism of co-factors encompassed pathways like “Porphyrin and Chlorophyll Metabolism”, “Pantothenate and CoA Biosynthesis” and “Nicotinate and Nicotinamide Metabolism” (Fig. 4, Supplementary Dataset File 1). Importantly, our analysis also revealed a strong connection between the enrichment of lipid metabolism genes (identified through transcriptomics), and the presence of differentially abundant metabolites like formate, lactate, and pantothenate (Fig. 4b). These metabolites are integral to the vitamin and co-factor metabolism pathways (Fig. 4b).

### HsFN3K interactome is enriched for metabolic pathways related to fatty acid and pyruvate

Next, we sought to elucidate the HsFN3K interactome to gain deeper insight into the enriched pathways identified from the transcriptomics and metabolomics datasets. We performed immunoprecipitation (IP) experiments followed by Liquid Chromatography and tandem Mass Spectrometry (LC-MS/MS). IP was performed using HsFN3K antibody on both the WT and FN3K KO HepG2 cells. Proteins that were present only in the WT HepG2 IP samples or had normalized Label Free Quantitation (LFQ) abundance ratio (WT/KO) greater than 1.5 were identified as potential interaction partners of HsFN3K (Supplementary Dataset File 1). For a broader analysis, we merged the list of interacting partners from three independent experiments and identified a total of 205 potential interacting partners.

**Fig. 5:**
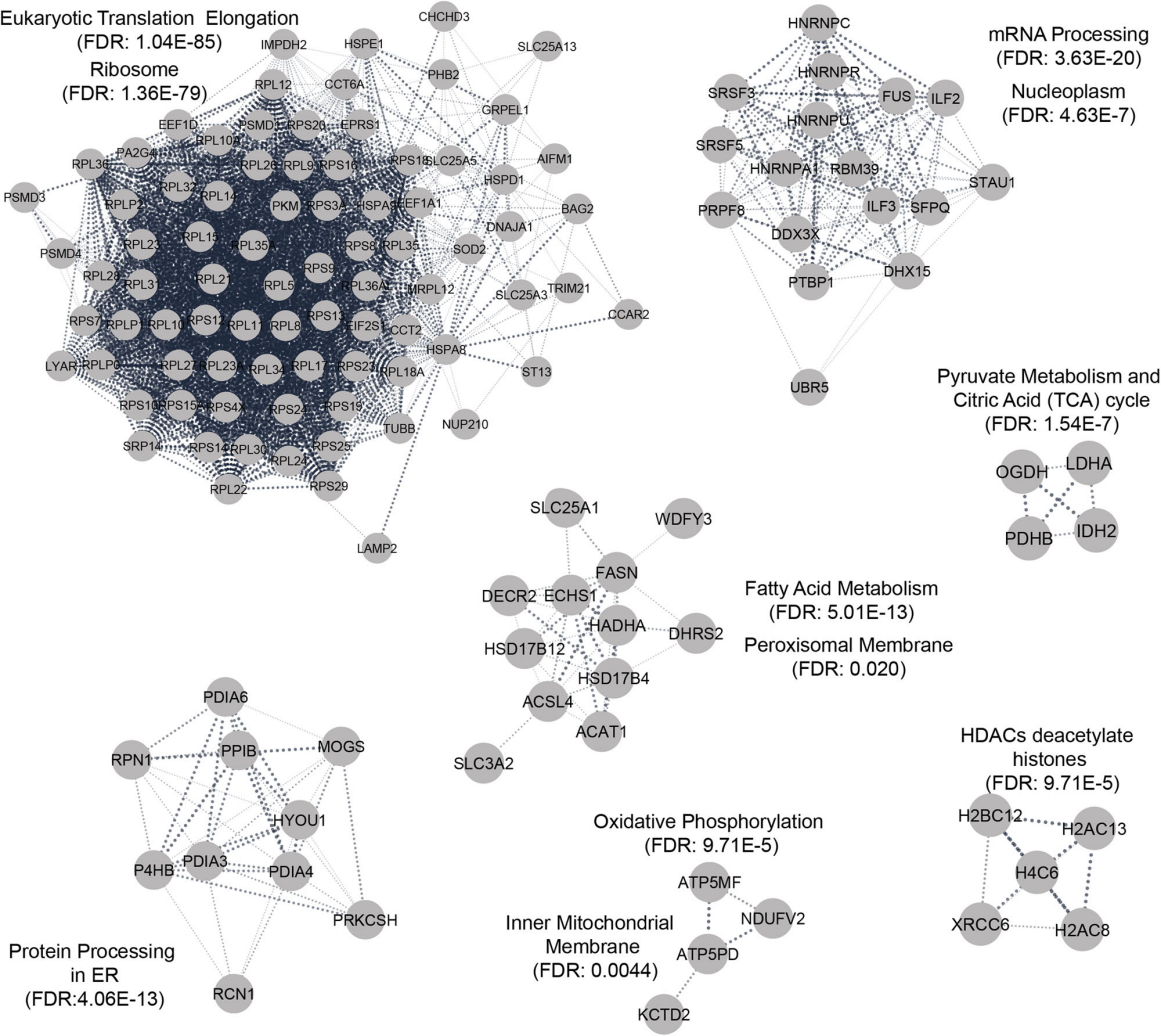
HsFN3K interactome is enriched in metabolic pathways such as fatty acid and pyruvate metabolism. HsFN3K interacting partners were identified through immunoprecipitation (IP) using anti-HsFN3K antibody followed by LC-MS/MS. Proteins with Label Free Quantitation (LFQ) normalized abundance ratio between WT and KO greater than 1.5 were identified as a potential interacting partner. StringApp in Cytoscape was used to define the edges with threshold of 0.4 confidence. Clustering was performed using MCL algorithm with granularity parameter set to 3.

We analyzed the interacting partners using Cytoscape StringApp framework with medium confidence cutoff value of 0.4 and no additional interactors. To reduce the complexity of the network, we clustered the nodes in the network using Markov CLustering Algorithm (MCL)^29^ with granularity set to 3. Functional and pathway enrichments were then performed on the distinct clusters (Fig. 5). Two of the largest clusters were related to translation elongation and mRNA processing which are localized to Ribosome and Nucleoplasm respectively (Fig. 5). Interestingly, protein processing in endoplasmic reticulum (ER) was also enriched and notably included several protein disulfide isomerases such as protein disulfide isomerase (PDI) family A member 3/4/6 (PDIA3/4/6) and prolyl 4-hydroxylase subunit beta (P4HB aka PDIA1) (Fig. 5). PDI catalyzes isomerization of disulfides in proteins for proper folding^30^ (Fig. 5).

Additional partners of HsFN3K were enriched for metabolic pathways, particularly within fatty acid and pyruvate metabolism (Fig. 5). FASN^31^, responsible for the de novo synthesis of fatty acids, and Acyl-CoA Synthetase Long Chain Family Member 4 (ACSL4)^32^, crucial for converting fatty acids into fatty acyl-CoA esters for use in pathways such as beta-oxidation, were upregulated in FN3K KO HepG2 cells. These enzymes, identified as interacting partners, underscore the role of HsFN3K in fatty acid metabolic pathways.

LDHA^33^, responsible for converting pyruvate to lactate within the pyruvate metabolism pathway in the cytosol, emerged as another significant interacting partner of HsFN3K (Fig. 5). Moreover, we also identified enrichment of pathways such as oxidative phosphorylation within the inner mitochondrial membrane and histone deacetylation, predominantly orchestrated by Histone Deacetylases (HDACs) in the nucleus. These findings emphasize HsFN3K’s extensive impact not just on metabolic pathways but also on regulatory mechanisms within distinct sub-cellular compartments.

### Integrative transcriptomics and interactomics analyses indicate enrichment of NAD(P) binding and thiolase-like domains

**Fig. 6:**
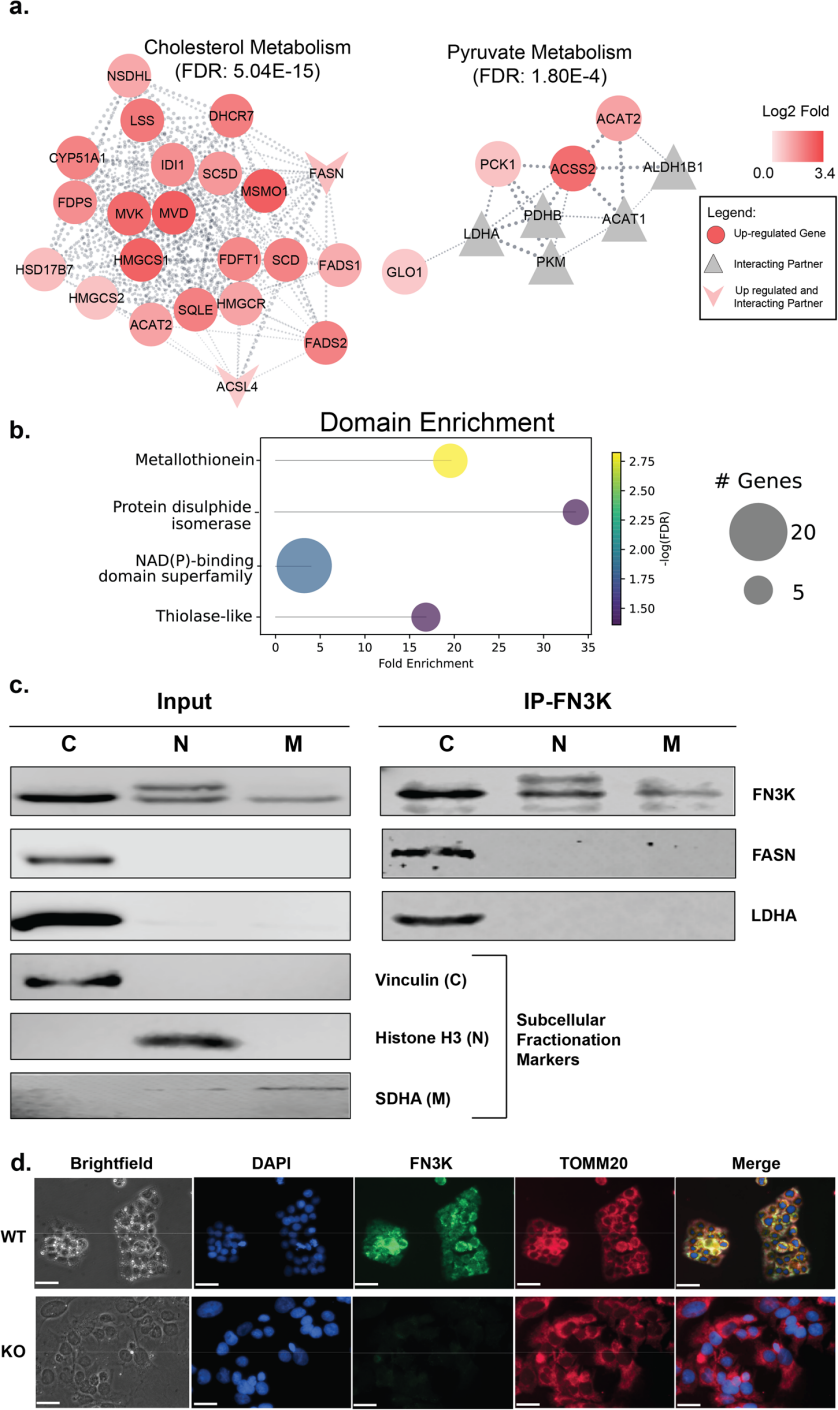
Integrative enrichment analysis of the transcriptomics and interactomics datasets using Cytoscape Stringapp framework. **a** DEGs identified from comparative transcriptomics are represented by circles, with upregulated and downregulated genes colored in red and blue, respectively. Interaction partners discovered through the Immunoprecipitation (IP) experiments are represented by triangles and colored gray. Proteins identified in both transcriptomic and interactomics data sets are represented as V symbols and colored based on their expression levels. **b** Lollipop graph showing domain enrichment on the integrative network. **c** Western blot analysis of subcellular fractionation of HepG2 cell lysate. Subcellular fractionation was followed by immunoprecipitation with anti-HsFN3K antibody on protein A/G agarose beads and immunoblotted for proteins as indicated. C: Cytoplasm; N: Nuclear; M: Mitochondria. Full Blots are shown in Supplementary Figure 3a, b. **d** Immunofluorescence (IF) with anti-HsFN3K (green) antibody on WT and FN3K KO HepG2 cells. DAPI (blue) and Tomm20 (red) were used as nuclear and mitochondrial markers respectively. The length of the scale bar is equal to 32 µM.

To better understand the complementary datasets of transcriptomics and interactomics, we performed pathway enrichment on the combined dataset using the Cytoscape StringApp framework. The combined network allowed us to expand on some of the enriched pathways and gain better insight into the interplay between the two datasets. Both FASN and ACSL4, identified in both the datasets, are not only involved in fatty acid metabolism but are also connected to cholesterol metabolism (Fig. 6a). Similarly, pyruvate metabolism was identified as an enriched pathway not only based on the interactomics dataset (Fig. 5) but also from the joint pathway analysis combining metabolomics and transcriptomics datasets (Fig. 4a). In the integrative network combining transcriptomics and interactomics datasets, we find that it not only includes enzymes such as LDHA, acyl-CoA synthetase short chain family member 2 (ACSS2), and acetyl-CoA acetyltransferase 2 (ACAT2) but also additional enzymes including phosphoenolpyruvate carboxykinase 1 (PCK1) (Fig. 6a). The upregulated PCK1 gene is the major indication of gluconeogenesis, a reverse process of glycolysis^34^.

In the integrative network, we also find enrichment of protein domains. While metallothioneins and protein disulfide isomerase domains enrichment can be identified individually from the transcriptomics and interactomics datasets respectively, the NAD(P) binding domain superfamily and thiolase-like domains were only enriched in the integrative network (Fig. 6b). NAD(P) binding domain superfamily proteins bind nicotinamide dinucleotide (NAD) or its phosphorylated form (NADP). These are co-factors that exist as redox couples (NAD + /NADH and NADP + /NADPH) in cells to serve distinct functions^35^. While NAD + /NADH is involved in cellular energy metabolism, NADP + /NADPH is involved in redox balance and fatty acid and nucleic acid synthesis^35^. On the other hand, Thiolase-domain proteins are involved in formation of acetoacetyl coA from acetyl-coA^36^.

The interactomics data hinted that HsFN3K might localize to various sub-cellular compartments, prompting us to delve deeper into this aspect through sub-cellular fractionation (SCF) and western blotting. The SCF of HepG2 cell lysates, followed by immunoblotting using an HsFN3K antibody revealed HsFN3K’s presence in the nucleus, mitochondria, and cytoplasm (Fig. 6c, Input). Notably, two distinct bands were observed in the nuclear fraction (Fig. 6c, Input). To determine if either of the bands was non-specific, we performed SCF on FN3K KO HepG2 cells. The top band in the nuclear fraction was also present in the FN3K KO cells, suggesting it was non-specific (Supplementary Fig. 3a). The fractionation was validated using loading markers: Vinculin for the cytoplasm (C), Histone H3 for the nucleus (N), and SDHA for the mitochondria (M) (Fig. 6c). In addition, we employed Immunofluorescence (IF) as a complementary technique to examine the localization of HsFN3K in HepG2 cells, using FN3K knockout (KO) cells as a negative control (Fig. 6d). We observed that, phenotypically, the nuclei of FN3K KO cells, stained with DAPI, appear larger than those in the wild type (WT) cells and overall, the KO cells do not form clumps as seen in WT HepG2 cells. Importantly, the anti-HsFN3K antibody did not produce any staining in the FN3K KO cells. Most notably, we detected co-localization of HsFN3K with TOMM20, a mitochondrial marker, evidenced by the emergence of yellow colors in the merged images (Fig. 6d), highlighting the mitochondrial presence of HsFN3K.

By leveraging the SCF lysates, we conducted IP with the anti-HsFN3K antibody to further investigate the interactions of HsFN3K with FASN and LDHA involved in fatty acid and pyruvate metabolic pathways respectively. FASN, previously identified as upregulated in the KO and a potential HsFN3K partner, was found co-immunoprecipitated in the cytoplasmic fraction (Fig. 6c, IP-FN3K). Contrary to our RNA sequence data, we find that the protein levels of FASN is reduced in the KO cells relative to WT (Supplementary Fig. 3b). Similarly, LDHA also co-immunoprecipitated with HsFN3K in the cytoplasmic fraction (Fig. 6c, IP-FN3K), reinforcing their interaction. Crucially, neither protein co-immunoprecipitated with agarose beads alone (Supplementary Fig. 4a), indicating specific interactions.

To investigate the specificity of the interaction between LDHA and HsFN3K, we treated the HepG2 cytoplasmic fraction with 6X Histidine-tagged HsFN3K, both wild type and mutants, purified from *E. coli*. We mutated residues in different structural and functional regions such as in nucleotide binding (F39V), divalent metal ion binding (D234A), and substrate binding (W219A) as well as redox insensitivity (C24A). After enriching HsFN3K using cobalt talon beads and conducting immunoblotting for LDHA, we observed that, although C24A showed the highest level of enrichment, the C24A mutation, along with the W219A mutation, impaired HsFN3K’s ability to interact with LDHA (Supplementary Fig. 4b). This suggests that these specific residues are crucial for the interaction with LDHA.

### HsFN3K binds to NAD related compounds specifically and in a metal dependent manner

**Fig. 7:**
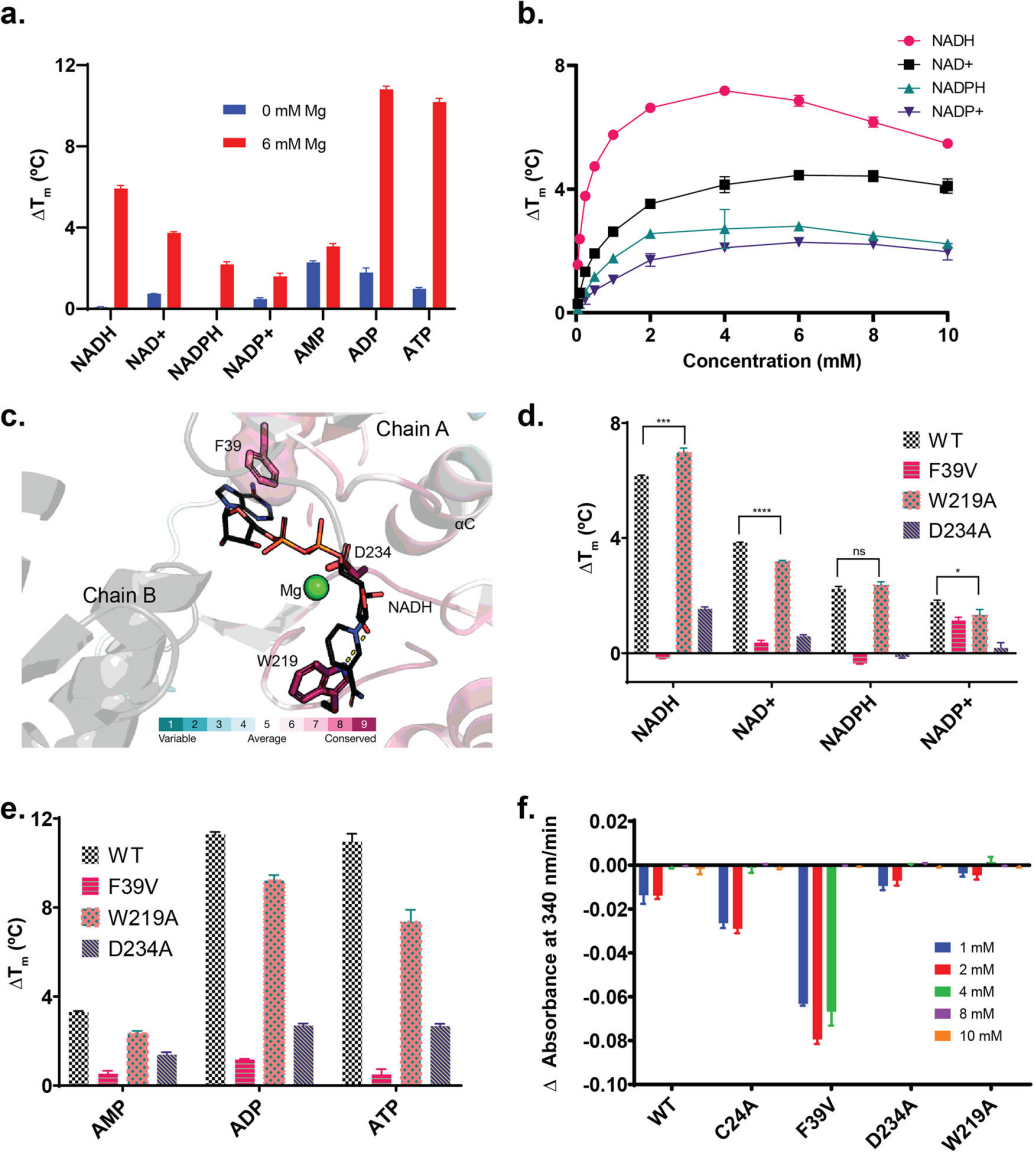
Interaction of Human FN3K (HsFN3K) and mutants with NAD compounds. **a** A bar chart showing shift in melting temperatures (ΔT_m_) for HsFN3K WT with 0 mM (blue) or 6 mM (red) magnesium (Mg). All tested compounds had a concentration of 5 mM. **b** Changes in ΔT_m_ of HsFN3K WT with 10 mM Mg at varied NAD compound concentrations ranging from 0.050 mM to 10 mM. **c** Homology model depicting the dimer of HsFN3K with NADH and Mg, utilizing Acedock and Metal Ion Binding (MIB) server, respectively. *A. thaliana* FN3K crystal structure (PDB ID: 6OID) was used as a template. The degree of conservation of the residues was evaluated and color-coded using the ConSurf server. **d** Bar chart showing ΔT_m_ for HsFN3K WT and the mutants in the presence of various NAD compounds. Asterisks (*) indicate values that were significantly different from the W219A mutant as compared to the WT (p < 0.05, Student’s t-test). **e** Bar chart showing ΔT_m_ for HsFN3K WT and the mutants in the presence of different nucleotides. **a**, **b**, **d**, **e** Mean ΔT_m_ values ± s.d. were calculated from 3 independent experiments (N = 3) and were calculated by subtracting the control T_m_ value (Apo). **f** Inhibition of HsFN3K WT and mutants’ activity by different concentrations of NADH. Pyruvate Kinase/Lactate dehydrogenase assay with Ribuloselysine as substrate was used. Mean values ± s.d. were calculated from 3 independent experiments (N = 3).

Next, based on the enriched pathways and metabolites, we conducted a screening to assess the binding of purified Wild Type (WT) HsFN3K to a select group of compounds, utilizing the Differential Scanning Fluorimetry (DSF) assay. While compounds such as lactic acid, creatine, nicotinamide riboside, and oxidized glutathione exhibited no discernible changes in thermal stability, both CoA and reduced glutathione displayed destabilizing effects regardless of the presence or absence of magnesium (Supplementary Fig. 5a). Notably, both reduced glutathione and Dithiothreitol (DTT) induced significant destabilization of the WT enzyme when compared to the C24A P-loop cysteine mutant (P values: 1.7E-4; 2.0E-6) (Supplementary Fig. 5b). Moreover, various NAD compounds exhibited distinct effects on stabilizing the WT HsFN3K, primarily when magnesium was present. Although the stabilities were relatively lower compared to ADP or ATP, they surpassed those of AMP (Fig. 7a). Interestingly, the reduced forms (NADH/NADPH) demonstrated greater thermal stability than their oxidized counterparts (Fig. 7a). Notably, among the NAD compounds, NADH exhibited the highest thermal stability. Subsequently, we evaluated the specificity of this interaction by subjecting the enzyme to DSF at varying concentrations of the NAD compounds. Indeed, all NAD compounds displayed saturation kinetics, suggesting a specific binding affinity to the enzyme (Fig. 7b). Notably, at sub-millimolar concentrations, NADH exerted the most pronounced stabilizing effect on HsFN3K when compared to other NAD compounds.

### The ATP binding pocket is a likely binding site for NADH

To better understand how NAD compounds bind to HsFN3K, we conducted docking experiments using Acedock^37^. Since NADH exhibited the highest stabilization effect in our DSF assays, we proceeded to dock the NADH molecule onto the homology model of HsFN3K. Leveraging the crystal structure of the plant homolog from *A. thaliana* FN3K (AtFN3K) (PDB ID: 6OID) as a template, which we had previously demonstrated forms a disulfide-linked dimer^16^, we aimed to shed light on the binding mode of HsFN3K. Additionally, we introduced a magnesium ion into the homology model using the Metal Ion-Binding webserver^38^, given that our DSF assays indicated magnesium dependency for NADH binding. Since NADH encompasses the adenine dinucleotide phosphate (ADP) moiety, we executed scaffold docking based on the ADP molecule’s placement within the AtFN3K crystal structure.

Among the diverse predicted binding modes for NADH, the top-scoring mode revealed the involvement of highly conserved aromatic residues, F39 and W219, within the ADP binding pocket (Fig. 7c). The nicotinamide moiety of NADH mediates hydrogen bonds along with CH-π interactions with W219 (Fig. 7c), while the adenine moiety engages in π-π interaction with F39. Notably, F39 in HsFN3K corresponds to F47 in AtFN3K, which partakes in a π-π interaction with the adenine ring of the ADP molecule within the crystal structure (PDB ID: 6OID)^16^.

To delve deeper into the specificity of the interaction between NAD compounds and HsFN3K, we performed site-directed mutagenesis guided by our docking findings. Subsequently, we conducted DSF assays on the mutant proteins. Specifically, we substituted the aromatic F39 and W219 with aliphatic valine (F39V) and alanine (W219A) residues, respectively. Additionally, we introduced an alanine mutation (D234A) for the metal chelating D234 as a control.

Among the three mutants, F39V led to the complete elimination of binding to NADH and NADPH, while retaining detectable thermal stabilities for NAD+ and NADP+ (Fig. 7d), along with the nucleotides (AMP/ADP/ATP) (Fig. 7e). Interestingly, the W219A mutant displayed subtle alterations in thermal stability between the reduced and oxidized states of NAD compounds. As compared to the WT enzyme, W219A mutant exhibited increased stability in the presence of reduced NAD compounds (NADH/NADPH) but diminished thermal stability when exposed to the corresponding oxidized forms (NAD + /NADP + ). Although nuanced, these stability changes were statistically significant for NADH (P value: 7.0E-4) and NAD+ (P value: 1.4E-5) (Fig. 7d). Intriguingly, the W219A mutation also reduced thermal stability for all tested nucleotides (Fig. 7e). As for the D234A mutant, detectable thermal stability was observed for NADH and NAD + , while the phosphorylated forms NADPH and NADP+ did not exhibit notable stability changes (Fig. 7d). Given NADH showed the most thermal stability among the NAD compounds, we tested the role of NADH on HsFN3K activity. Interestingly, we find that 4 mM NADH inhibits HsFN3K activity completely for both WT and C24A mutants, but not for the F39V mutant. This suggests a reduced sensitivity to NADH inhibition by the F39V mutant in a concentration-dependent manner (Fig. 7f). Moreover, C24A is stabilized by both NADH and NAD + , with NADH showing a higher thermal stability compared to WT, particularly in the absence of DTT (Supplementary Fig. 5c).

## Discussion

**Fig. 8:**
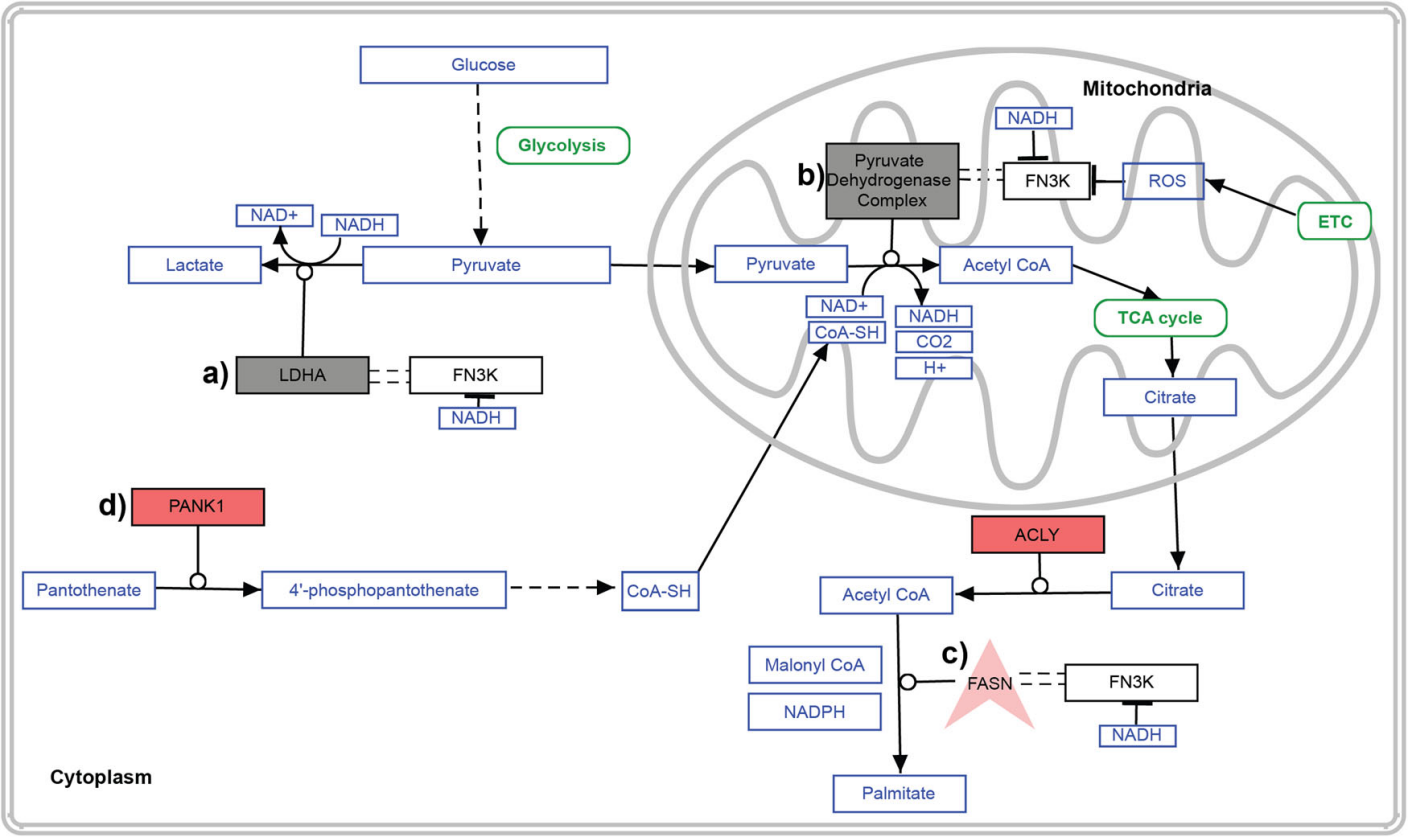
Model of HsFN3K cellular regulation and functions. Summary of the enriched pathways based on integrative-omics studies. Red Rectangle: Upregulated differentially expressed genes; Gray Rectangle: FN3K Interacting Partner, and Red V shape: FN3K partner and upregulated differentially expressed genes. PathVisio3^58^ was used to generate the figure.

We leveraged the FN3K CRISPR KO in HepG2 cells and performed the first comprehensive study to investigate the function of FN3Ks in a larger pathway context. Our multi-omics analyses and biochemical assays have illuminated FN3K’s potential role in lipid metabolism—specifically in the metabolism of cholesterol and fatty acids. This role is intricately linked to carbon (via pyruvate metabolism) and co-factor metabolism pathways (involving CoA and nicotinic acid/nicotinamide), providing a clearer picture of FN3K’s metabolic involvement (Figs. 3–6, 8**)**.

A particularly interesting finding from our study is the specific interaction of HsFN3K with NAD compounds in a metal-dependent manner, with its activity notably inhibited by NADH (Fig. 7). While there have been no previous studies investigating the binding of FN3Ks to NAD compounds, an unbiased NAD interactome study using clickable, photoaffinity labeling identified HsFN3KRP in one of its replicates^39^. Notably, the study was done with HEK293T cell lysates where HsFN3K expression is low (2.2 nTPM as compared to 51 nTPM for HsFN3KRP)^40^. To our knowledge, AMP-activated protein kinase (AMPK) is the only kinase fold enzyme previously reported to bind NAD compounds with NADH inhibiting AMPK activity and NAD+ activating it^41^.

Additionally, our study observed a reduction in 5’-AMP-activated protein kinase subunit beta-2 (PRKAB2) levels, the non-catalytic subunit of AMPK, in FN3K KO cells, indicating potential areas for deeper investigation in future research.

Our in-depth interactomics analysis greatly expands the known protein-protein interaction (PPI) network of HsFN3K, which was previously confined to just two partners in the IntAct database^42^ and ten in BioGRID^43^. We have identified 205 potential interaction partners of HsFN3K that participate in key cellular functions in different sub-cellular compartments including mitochondria and nucleus (Fig. 5). Despite the lack of sub-cellular localization signal, immunohistochemistry studies from the Human Protein Atlas^15^ and from this study (Fig. 6d) have demonstrated the localization of HsFN3K within mitochondria in HepG2 cells. Our research corroborates and extends these findings, revealing HsFN3K’s presence in the nucleus and mitochondria, in addition to the cytoplasm (Fig. 6c). This wide-ranging localization supports the enzyme’s role in a variety of distinct but interconnected cellular pathways. For example, in the cytoplasm, LDHA catalyzes the conversion of pyruvate to lactate (Fig. 8a), whereas in mitochondria, Pyruvate dehydrogenase B (PDHB), a component of the pyruvate dehydrogenase complex, converts pyruvate into acetyl CoA (Fig. 8b). Acetyl CoA is subsequently used by FASN for palmitate synthesis in the cytoplasm (Fig. 8c), with CoA being synthesized from pantothenate (Fig. 8d). Intriguingly, we observed an upregulation of Pantothenate Kinase 1 (PANK1) and a decreased abundance of pantothenate in FN3K KO cells. These enzymatic activities, driven by the conversion of NAD redox couples (Fig. 8), suggest that HsFN3K’s binding to various NAD compounds—and its inhibition by NADH—indicates changes in these redox couple ratios could critically regulate HsFN3K activity within cells.

In cells, two out of four major redox couples are related to NAD compounds: NAD + /NADH and NADP+ and NADPH. The remaining two are part of the antioxidant systems and include glutathione (GSSG/GSH) and thioredoxin (Trx(SH)2/TrxSS)^44^ couples. NADP + /NADPH redox couple is critical to the antioxidant system as it donates electrons to glutathione and thioredoxin systems^44^. Both these systems play a pivotal role in neutralizing reactive oxygen species (ROS) produced by the electron transport chain (ETC) in mitochondria^45^. ROS can alter redox-active cysteines in proteins, leading to sulfenylated cysteines or disulfide bridges, which in many cases initiate oxidative stress response signaling within cells^46^.

Our previous research demonstrated that under oxidative stress, HsFN3K forms disulfide-linked dimers through the P-loop cysteine (C24)^16^. Additionally, other studies have observed sulfenylation at the cysteine residue in both HsFN3K and HsFN3KRP^47^. The presence of HsFN3K in mitochondria, coupled with the enrichment of oxidative stress response pathways and metabolites, including glutathione, further underscores the connection between HsFN3K and oxidative stress response. Under conditions that generate ROS, HsFN3K may undergo covalent modification at the P-loop cysteine, influencing its activity and interactions with other proteins (Fig. 8). Our findings suggest that mutations in the P-loop cysteine can disrupt the interaction with LDHA (Supplementary Fig. 4b). Furthermore, HsFN3K’s interaction with several protein disulfide isomerases (PDIs) suggests that FN3K dimerization via disulfide bonds could be modulated in the ER (Fig. 5). Since NRF2, the master regulator of oxidative stress response, is deglycated by HsFN3K^17^, it is possible that HsFN3K KO alters oxidative stress response and redox balance through the NRF2 pathway. Similarly, due to oxidative stress being a major risk factor for chronic obstructive pulmonary disease (COPD), others have studied expression levels of FN3Ks in lungs between control and COPD patients^48^. Additionally, an exome-wide association study has highlighted HsFN3KRP as a potential candidate gene for longevity^7^. Collectively, these findings, along with our research, underscore the importance of further exploring the FN3K family’s roles in both health and disease.

## Methods

### FN3K expression in tissues and cancer cells

mRNA expression levels of human Fructosamine 3 kinase (FN3K) and FN3K-related protein (FN3K-RP) across various tissues were obtained from the Human Protein Atlas (HPA), using the “rna_tissue_consensus.tsv” file. This data, including the consensus normalized expression (nTPM), is based on HPA version 23.0 and Ensembl version 109.

The levels of HsFN3K RNA expression across various cancer types were arranged by median expression values. The figure was produced using cBioPortal. Unit for mRNA expression levels: RSEM (Batch normalized from Illumina HiSeq_RNASeqV2) (log2(value + 1)).

### RNA-seq sample preparation

FN3K KO was generated in HepG2 cells (bought from American Type Culture Collection (ATCC) HB-8065) as described previously^16^. HepG2 (WT and KO) cells were cultured on 6 cm plates in triplicate to 70–80% confluency in Eagle’s Minimum Essential Media (EMEM, ATCC) containing 10% Fetal Bovine Serum. Cells were washed with PBS and harvested. The cell pellet obtained was shipped on dry ice to Novogene Corporation Inc (1007 Slater Road, Suite 140, Durham, NC 27703) for RNA extraction and RNA seq analysis.

### Immunoprecipitation and Mass Spectrometry sample preparation

HepG2 (WT and KO) cells were cultured on 10 cm plate to 80–90% confluency. Cells were washed with PBS and lysed in 1 ml of lysis buffer (50 mM Tris-HCl, pH 7.4, 150 mM NaCl, 1% Triton X-100, 1 mM EDTA, 10% glycerol and protease inhibitor cocktail). Cell lysate was spun down at 12000 rpm, and supernatant was subjected to immunoprecipitation with rabbit FN3K Polyclonal Antibody (1:200 dilution) (Invitrogen Catalog # PA5–28603) and 40ul slurry of protein A/G plus agarose beads (Pierce 20423) at 4 C for 18 h. Beads were washed 3 x lysis buffer, and bound proteins were eluted in 1x Laemmli SDS sample buffer. Proteins were resolved on 10% SDS-PAGE gel by running gel for 10 min (avoid running gel to full to minimize gel amount). The whole lane was cut and sent for trypsinization and MS analysis.

In-gel trypsin digestion protocol was as follows. First, the gel bands were sliced into small pieces, and then rinsed with 50% acetonitrile/20 mM ammonium bicarbonate ( ~ pH7.5–8) twice. The gel pieces were dehydrated by adding 100% acetonitrile and dried out by a SpeedVac. A various amount of Trypsin solution (0.01 µg/µL in 20 mM ammonium bicarbonate) was added until the gel pieces totally absorbed the Trypsin solution. The tubes were placed in an incubator at 37 °C overnight. The tryptic peptides were extracted from gel pieces by incubating with 50% acetonitrile/0.1% formic acid twice. The extracts were dried down by a SpeedVac.

Mass spectrometry analyses were performed on a Thermo-Fisher LTQ Orbitrap Elite Mass Spectrometer coupled with a Proxeon Easy NanoLC system (Waltham, MA) located at Proteomics and Mass Spectrometry Facility at the University of Georgia.

The enzymatic peptides were loaded into a reversed-phase column (self-packed column/emitter with 200 Å 5 µM Bruker MagicAQ C18 resin), then directly eluted into the mass spectrometer. Briefly, the two-buffer gradient elution at the flow rate 450 nL/min (0.1% formic acid as A and 99.9% acetonitrile with 0.1% formic acid as B) starts with 0% B, holds at 0% B for 2 minutes, then increases to 30% B in 50 minutes, to 50% B in 10 minutes, and to 95% B in 10 minutes.

The data-dependent acquisition (DDA) method was used to acquire MS data. A survey MS scan was acquired first, and then the top 10 ions in the MS scan were selected for following CID MS/MS analysis. Both MS and MS/MS scans were acquired by Orbitrap at the resolutions of 120,000 and 15,000, respectively.

Data were analyzed using Xcalibur software (version 2.2, Thermo Fisher Scientific). Protein identification and modification characterization were performed using Thermo Proteome Discoverer (version 1.4) with Mascot (Matrix Science 2.7) and Uniprot database. The spectra of possible modified peptides were inspected further to verify the accuracy of the assignments.

### RNA-Seq data analysis

We conducted RNA sequencing (RNA-Seq) on total RNA from the FN3K KO and the wild-type (WT) cell lines. RNA extraction, preparation of RNA library and transcriptome sequencing were conducted by Novogene Co., LTD. The obtained reads were then analyzed using the “new Tuxedo” package suite of tools^49^. HISAT2^23^ was first used to map the reads to the human reference genome separately for the KO and WT samples. This was performed using the default parameters. Then, the alignment was passed to StringTie^24^ for assembly and quantification of the transcripts. The assembled transcripts were then passed to Ballgown^25^ to perform statistical tests to identify differentially expressed transcripts between the WT and KO samples. We used Ballgown’s stattest function to perform a statistical comparison of the FPKM values for all assembled transcripts at the gene level and report the p-value and the fold change for genes that were significantly differentially expressed. Only genes with p-value < 0.05 and a log fold change > 0.2 were considered as significantly differentially expressed and used in subsequent analyses.

### Pathway enrichment analysis

We collected the UniProt IDs for i) all the genes that were found to be differentially expressed in the RNA-Seq analysis and ii) the genes that were identified as interacting partners using IP. This list was uploaded individually or merged together into Cytoscape^50^ and the STRING Cytoscape plugin^51^ was used to build a STRING-based network of all these genes. We then used the STRING plugin to perform an enrichment analysis on this network of genes to find enriched pathways. The enrichment analyses on transcriptomics and interactomics datasets was performed using stringApp which uses KEGG^52^, Reactome^53^, and Wiki pathways^54^. We used a redundancy cutoff of 0.2 within STRING to remove pathways that had a high overlap of genes. We further filtered this list to remove small pathways that had 5 or less genes with an FDR value below 0.05 (Supplementary Dataset File 1). For metabolomics, we used Metaboanalyst which uses KEGG and Human Metabolome database.

We used fold enrichment, a quantitative measure of the over-representation of a pathway, to rank order the obtained pathways. Fold enrichment is obtained by comparing the background frequency of total genes annotated for that pathway on humans to the actual genes that mapped to that same pathway from the list that was provided. Specifically, fold enrichment for each pathway was calculated using the following proportion:

$${\rm{Fold\; enrichment}}=({\rm{n}}/{\rm{M}})/({\rm{Nb}}/{\rm{Nt}})$$

where,

- n is the total number of genes from our list that match the pathway.
- M is the total number of genes in our list.
- Nb is the total number of background genes (total genes in that pathway) in humans.
- Nt is the total number of genes in humans (19566, for human genome used by the STRING database version 11.5).

### Differential Scanning Fluorimetry (DSF)

Approximately 7 µM of Human FN3K WT or mutants (C24A, F39V, D234A, W219) were mixed with 1:500 SYPRO ORANGE (Sigma) in buffer (20 mM HEPES pH 7.4, 150 mM NaCl, 5% glycerol). The nucleotides or nicotinamides were added in the presence or absence of 6 mM MgCl_2_ to make the final concentration of 5 mM. The solution was then heated gradually from 20 °C to 95 °C at the rate of 0.3 °C, and the fluorescence was monitored using the StepOne Plus Real-time PCR instrument. Data was analyzed using Prism.

### Homology modeling, docking and ConSurf Analysis

The crystal structure of the plant homolog from *A. thaliana* (PDB ID: 6OID) was used as a template for the homology model of HsFN3K. ADP was modeled in the pocket by aligning the crystal structure with the homology model. Magnesium ion was docked on the homology model using the Metal-Ion Binding (MIB)^38^ webserver. NADH was docked onto the homology model using scaffold docking with ADP as a template on the Acedock^37^ webserver. Chain A of the homology model was used as a query for the ConSurf^55^ server.

### Subcellular Fractionation, immunoprecipitation (IP) and Western Blot

HepG2 cells were cultured on a 10 cm dish until they reached 80–90% confluency. Subcellular fractionation was conducted following a protocol from “Subcellular Fractionation: A Laboratory Manual”^56^. Briefly, cells from a 10 cm dish were lysed in 550 μL ice-cold RSB buffer (10 mM Tris-HCl, pH 7.5, 10 mM NaCl, 1.5 mM MgCl_2_) for 10 minutes and subjected to dounce homogenization. The lysed cells were then transferred to a microcentrifuge tube, and 400 µL of 2.5x homogenization buffer (12.5 mM Tris-HCl, pH 7.5, 525 mM mannitol, 175 mM sucrose, 2.5 mM EDTA) was added. The mixture was centrifuged at 1300 g to pellet nuclei (the nuclear fraction), which was then washed with 1x homogenization buffer (5 mM Tris-HCl, pH 7.5, 210 mM mannitol, 70 mM sucrose, 1 mM EDTA) to remove contaminants. The supernatant was transferred to a new tube and subjected to a repeat 1300 g centrifugation to ensure all nuclei were removed. The final supernatant was then transferred to a new tube and centrifuged at 17000 g for 15 minutes to pellet the mitochondria (the mitochondrial fraction was washed with 1x homogenization buffer to eliminate contaminants). The remaining supernatant was designated as the cytosolic fraction and centrifuged at 17000 g to clear mitochondrial debris. Nuclear and mitochondrial fractions were resuspended in 1 mL RIPA buffer (50 mM Tris-HCl, pH 7.4, 150 mM NaCl, 1% NP-40, 0.1% SDS, 0.5% deoxycholate) and sonicated, while the cytosolic fraction was dialyzed in RIPA buffer using Amicon centrifugal filters. Protein concentrations in each fraction were determined using a BioTek Synergy plate reader, all the subcellular fractions were normalized to 1 mg/ml total proteins. Equal amounts of protein (400ug) were subjected to immunoprecipitation with anti-FN3K antibody (1:200 dilution) (Invitrogen, Catalog #: PA5–28603) and 20 µL agarose protein A/G plus beads (Pierce). After 16 hours of incubation at 4 °C, the beads were washed three times in RIPA buffer, and the bound proteins were eluted in 1x Laemmli buffer and resolved on 10% SDS-PAGE gels. Western blotting was carried out by transferring proteins onto a PVDF membrane using a semi-dry transfer system. Interacting proteins were detected using antibodies against fatty acid synthase (1:2000 dilution) (Invitrogen, Catalog #: MA-531490) and LDHA (1:1000 dilution) (Cell Signaling Technology, Catalog #: C4B5). Antibodies against Vinculin (1:1000 dilution) (Cell Signaling Technology, Catalog #: E1E9V), Histone H3 (1:5000 dilution) (Cell Signaling Technology, Catalog #: D1H2), and SDHA (1:1000 dilution) (Cell Signaling Technology, Catalog #: 5839 S) served as subcellular markers. Detection of bound antibodies was performed (chemiluminescence) using the LI-COR Odyssey M imaging system.

### Immunofluorescence

HepG2 WT and KO cells were fixed with 4% p-formaldehyde for 30 min and washed three times with 1XPBS followed by blocking with 3% normal goat serum for 1 h. Cells were incubated with 1ug/ml FN3K (1:50 dilution) (Invitrogen, PA5–66239) and Tomm20 (1:100 dilution) (Abnova, H00009804-M01) antibodies at 4 °C for 24 h. Cells were washed three times with 1XPBS and incubated with secondary antibodies, Alexa Fluor rabbit 488 (rabbit-FN3K, green) and Alexa Fluor mouse 555 (mouse-Tomm20, red) for 24 h. Secondary antibodies were removed by washing three times with 1XPBS and nuclear staining was performed with 1ug/ml DAPI (blue) for 5 min. Cells were given final wash with 1XPBS followed by visualizing under Zeiss Observer.D1 microscope for imaging.

### Interaction of LDHA with Purified 6X-Histidine Tag HsFN3K

25 µL of 50% Co^2+^ Talon beads were prepared by washing three times with Phosphate-Buffered Saline (PBS). To these prepared beads, 400 µL of the HepG2 cytosolic fraction (from 1 mg/ml total proteins) was added, followed by the addition of 45 µL of 1 mg/mL His-tagged (HsFN3K) (wild-type and mutants as indicated). After 16 hours of incubation at 4 °C, the beads were washed three times in RIPA buffer. Bound proteins were eluted in 1x Laemmli buffer and resolved on 10% SDS-PAGE gels. Western blotting was performed as described above using anti-LDHA (1:1000 dilution) (Cell Signaling Technology, Catalog #: C4B5) and anti-His Tag (1:6000 dilution) (Cell Signaling Technology, Catalog #: 27E8) antibodies.

### NADH inhibition assay

A mixture containing 10.0 µg (5 µL) of HsFN3K WT and its mutants, along with 50 mM Ribulose-N-α-Ac-lysine (5 µL) to reach a final concentration of 5 mM, was prepared. This mixture was then combined with 20 µL of a specially prepared solution comprising 150 µL of 5X Kinase Buffer (containing 160 mM HEPES at pH 7.4, 80 mM MgCl2, 1.2 M NaCl, and 40% v/v Glycerol), 15 µL of 250 mM Phosphoenolpyruvic acid (PEP), 45 µL of a PK/LDH mixture [ranging from 600 to 1000 units/mL for pyruvate kinase (PK) and 900 to 1400 units/mL for lactic dehydrogenase (LDH)], and 90 µL of H2O. To test inhibition, 10 µL of NADH stock at varying concentrations was added. The reaction was initiated with the addition of 10 µL of 2.5 mM ATP, achieving a final concentration of 0.5 mM ATP. The total volume for each reaction well was brought to 50 µL. Subsequently, the 96-well plate was placed into a Biotek Synergy H4 plate reader, where the absorbance at 340 nm was continuously monitored at 35 °C for two and a half hours. For storage, proteins were kept in Buffer D (comprising 25 mM HEPES, pH 7.4, 300 mM NaCl, and 10% v/v Glycerol), which was also employed as the mock buffer when necessary.

### Reporting summary

Further information on research design is available in the Nature Research Reporting Summary linked to this article.

### Supplementary information

Supplementary Figures

Reporting summary

Supplementary Dataset File 1

## Data Availability

All relevant data supporting the findings of this study are available within the article (and its supplementary information files). RNA expression data for the WT and the FN3K KO samples have been deposited in the Gene Expression Omnibus database under the accession code GSE242555.
