# Supplementary material for: Multi-omics reveals new links between Fructosamine-3-Kinase (FN3K) and core metabolic pathways
Source: NPJ Syst Biol Appl. 2024 Jun 3;10:64. doi: 10.1038/s41540-024-00390-0 (PMC11148063; doi:10.1038/s41540-024-00390-0)
Supplement: Supplementary file 1 — Supplementary Figures [file 41540_2024_390_MOESM1_ESM.pdf]

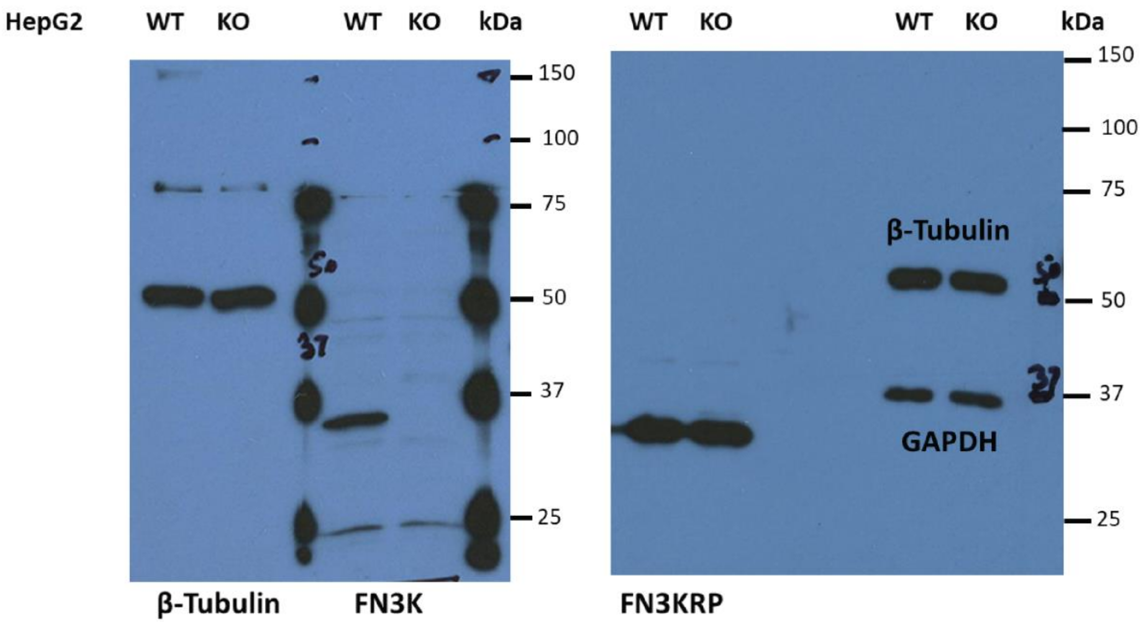

Supplementary Fig. 1. Full Western blots for blots shown in **Fig. 1c**. The blots originated from two separate SDS-PAGE gels and were cut into different strips to probe for FN3K, FN3KRP, and loading controls (β-Tubulin and GAPDH).

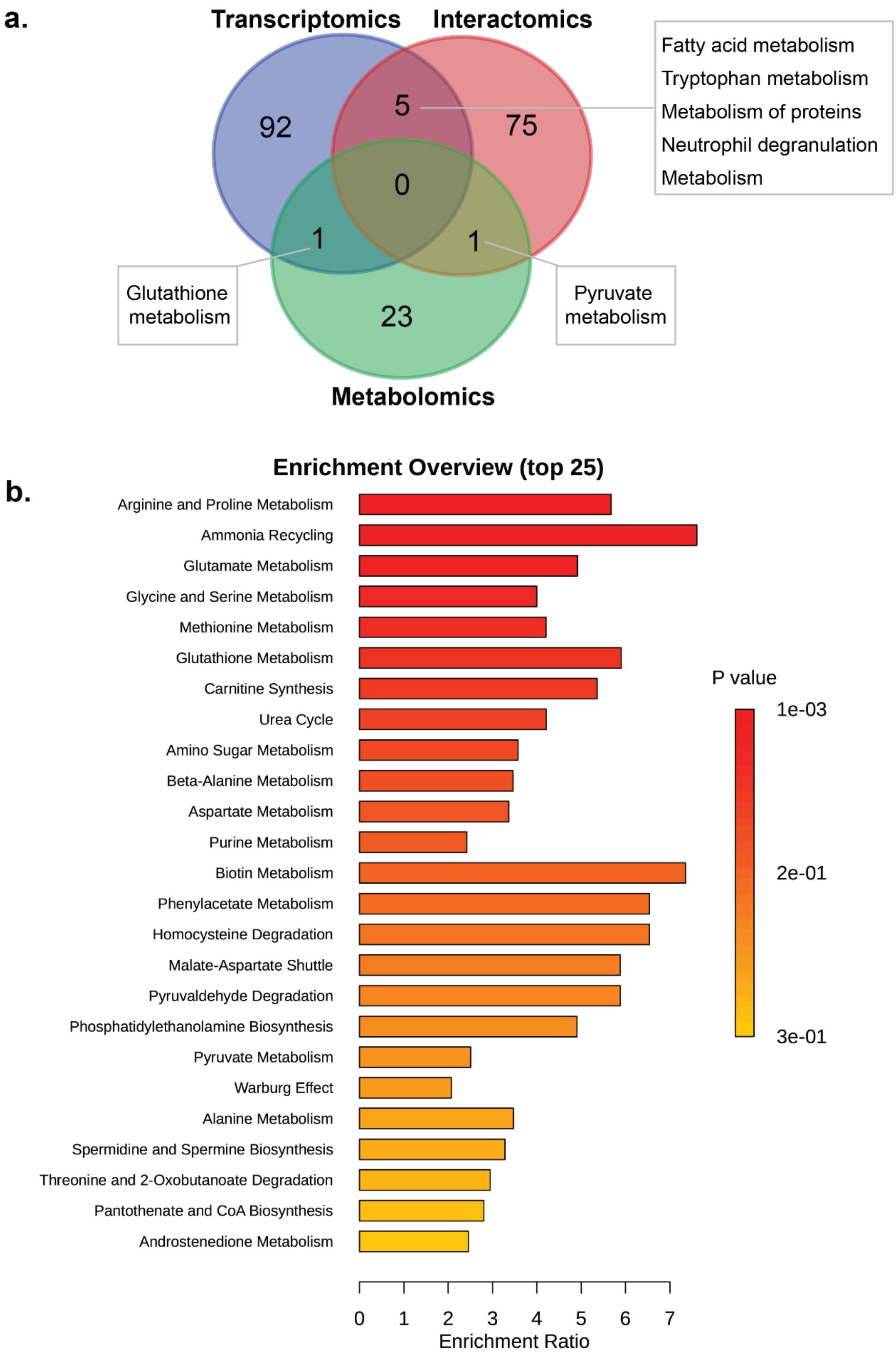
a.
Transcriptomics
Interactomics
92
5
75
0
1
1
23
Metabolomics
Fatty acid metabolism
Tryptophan metabolism
Metabolism of proteins
Neutrophil degranulation
Metabolism
Glutathione metabolism
Pyruvate metabolism
b.
Enrichment Overview (top 25)
Arginine and Proline Metabolism
Ammonia Recycling
Glutamate Metabolism
Glycine and Serine Metabolism
Methionine Metabolism
Glutathione Metabolism
Carnitine Synthesis
Urea Cycle
Amino Sugar Metabolism
Beta-Alanine Metabolism
Aspartate Metabolism
Purine Metabolism
Biotin Metabolism
Phenylacetate Metabolism
Homocysteine Degradation
Malate-Aspartate Shuttle
Pyruvaldehyde Degradation
Phosphatidylethanolamine Biosynthesis
Pyruvate Metabolism
Warburg Effect
Alanine Metabolism
Spermidine and Spermine Biosynthesis
Threonine and 2-Oxobutanoate Degradation
Pantothenate and CoA Biosynthesis
Androstenedione Metabolism
P value
1e-03
2e-01
3e-01
0 1 2 3 4 5 6 7
Enrichment Ratio

Supplementary Fig. 2. (a) Venn diagram showing common pathways between the three omics datasets. Numbers in the diagram indicate counts of Pathway description. **(b)** Summary Plot for Over Representation Analysis (ORA) for differentially abundant metabolites between WT and FN3K KO HepG2 cells using Metaboanalyst.

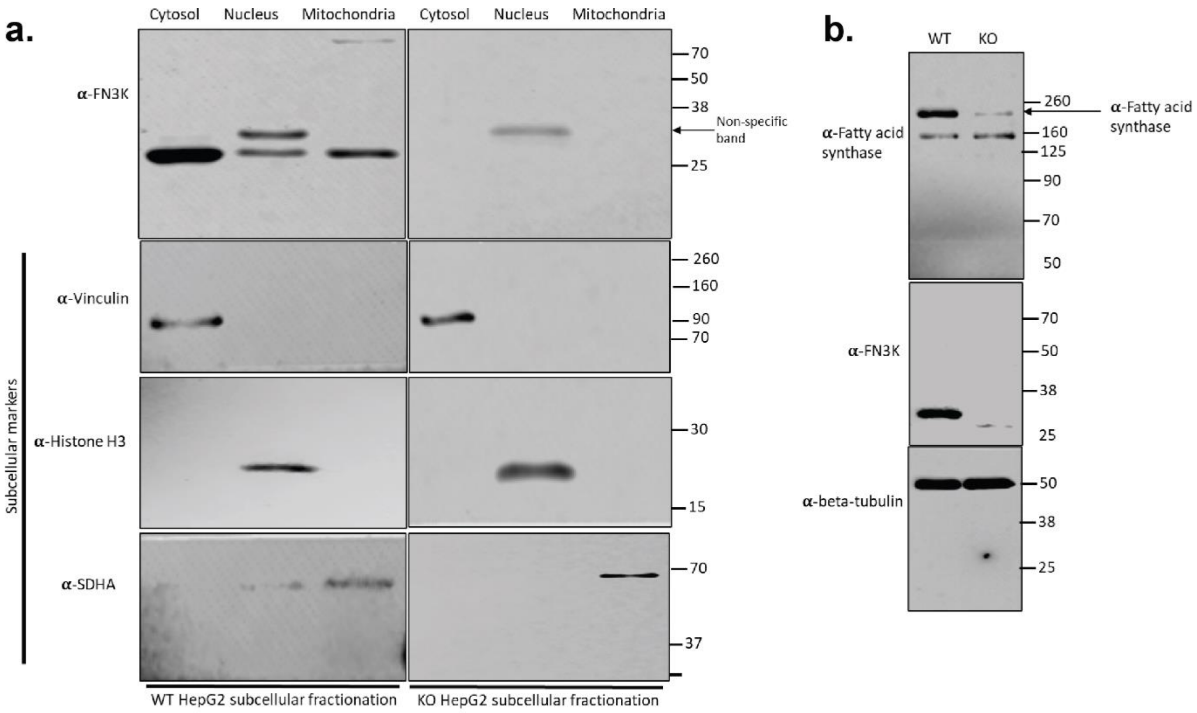

Supplementary Fig. 3. (a) Subcellular fractionation of WT and FN3K KO HepG2 cells immunoblotted with FN3K antibody. Subcellular markers: Cytoplasm: Vinculin; Nucleus: Histone; Mitochondria: SDHA. **(b)** Western blot showing levels of Fatty acid synthase and FN3K in the WT and FN3K KO HepG2 cells. Beta-tubulin as loading control.

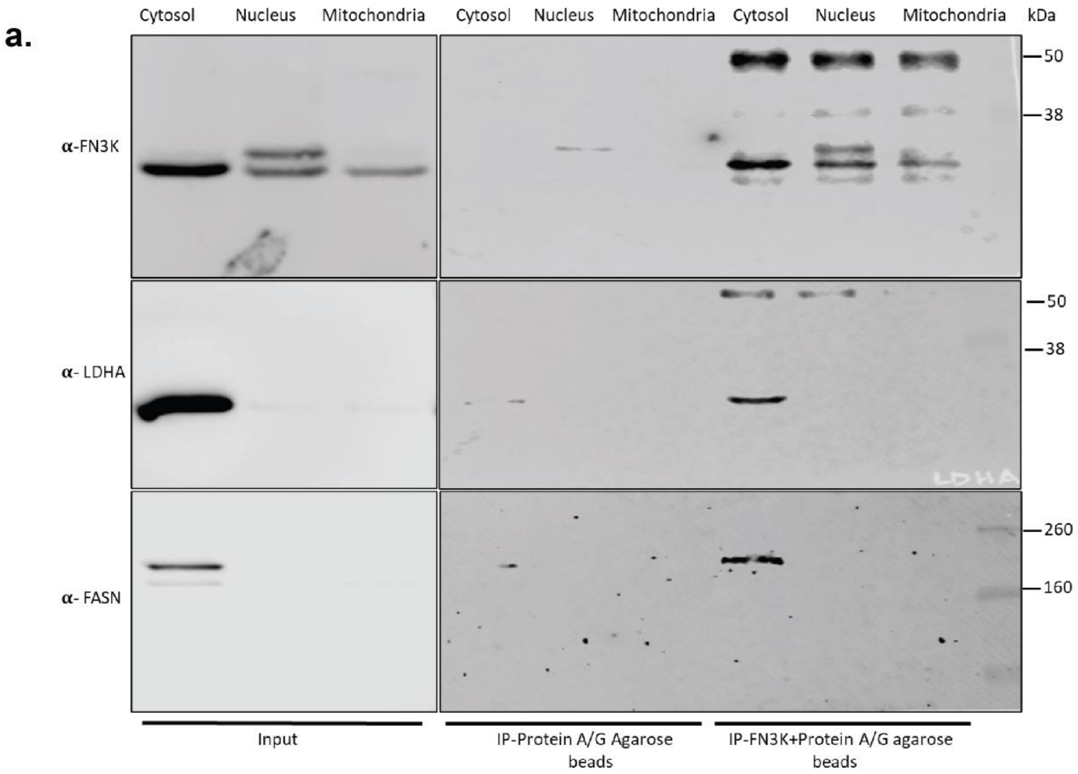

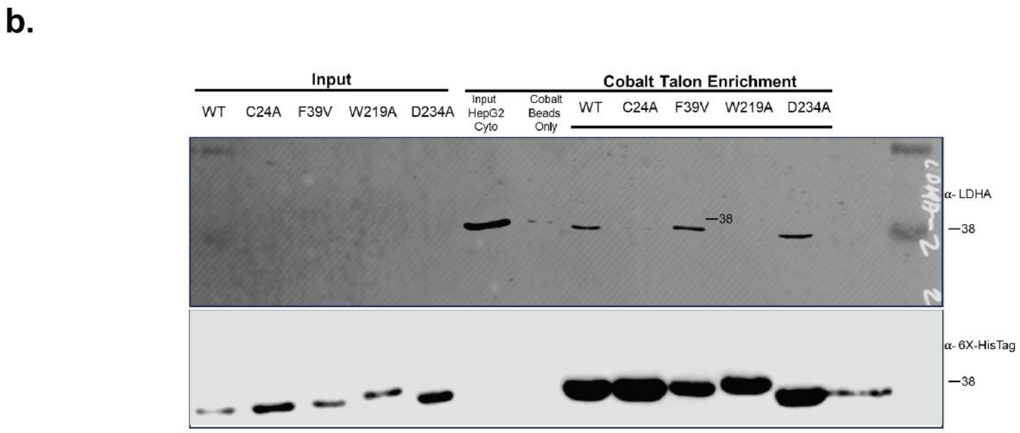

Supplementary Fig. 4. (a) Full Western blots for blots shown in Fig. 6c. **(b)** Western blots showing interaction of LDHA from HepG2 cytosolic fraction with purified 6X-Histidine tag HsFN3K and mutants. LDHA was coimmunoprecipitated with 6X HisTag HsFN3K differentially by WT and mutant proteins. Anti-6X-HisTag immunoblotting shows levels of HsFN3K in the input and enrichment. The blots are from the same experiment.

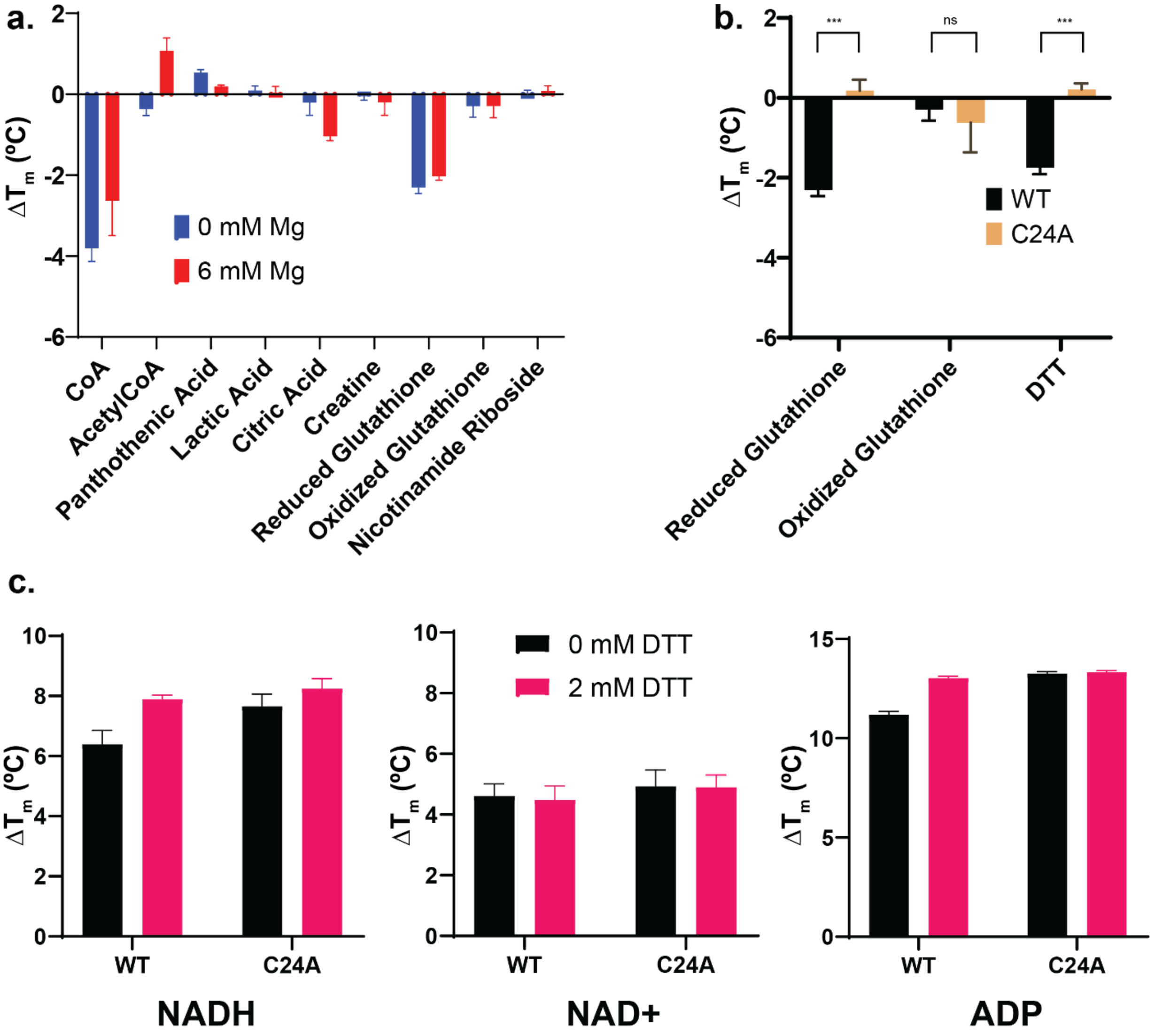

Supplementary Fig. 5. (a) A bar chart showing shift in melting temperatures (ΔT_m) for HsFN3K WT with 0 mM (blue) or 6 mM (red) magnesium (Mg) with different metabolites. **(b)** A bar chart showing shift in melting temperatures (ΔT_m) for HsFN3K WT (black) and C24A (yellow) with 2 mM of reduced and oxidized glutathione as well as DTT. Asterisks (*) indicate values that were significantly different from the C24A mutant as compared to the WT ($p<0.05$, Student's t-test). **(c)** Bar chart showing shift in melting temperatures (ΔT_m) for WT and C24A mutant with 5 mM NADH, NAD+, and ADP in the absence and presence of 2 mM DTT. 6 mM $MgCl_2$ was present. DTT: 1,4-Dithiothreitol. Mean ΔT_m values ± s.d. were calculated from 4 independent experiments (N=4).
