# Supplementary material for: Multi-omics reveals new links between Fructosamine-3-Kinase (FN3K) and core metabolic pathways
Source: NPJ Syst Biol Appl. 2024 Jun 3;10:64. doi: 10.1038/s41540-024-00390-0 (PMC11148063; doi:10.1038/s41540-024-00390-0)
Supplement: Supplementary file 2 — Reporting summary [file 41540_2024_390_MOESM2_ESM.pdf]

nature portfolio

Corresponding author(s): Natarajan Kannan

Last updated by author(s): 2024/04/15

Reporting Summary

Nature Portfolio wishes to improve the reproducibility of the work that we publish. This form provides structure for consistency and transparency in reporting. For further information on Nature Portfolio policies, see our Editorial Policies and the Editorial Policy Checklist.

Statistics

For all statistical analyses, confirm that the following items are present in the figure legend, table legend, main text, or Methods section.

n/a	Confirmed	
☐	☒	The exact sample size (*n*) for each experimental group/condition, given as a discrete number and unit of measurement
☐	☒	A statement on whether measurements were taken from distinct samples or whether the same sample was measured repeatedly
☐	☒	The statistical test(s) used AND whether they are one- or two-sided<br>*Only common tests should be described solely by name; describe more complex techniques in the Methods section.*
☒	☐	A description of all covariates tested
☐	☒	A description of any assumptions or corrections, such as tests of normality and adjustment for multiple comparisons
☐	☒	A full description of the statistical parameters including central tendency (e.g. means) or other basic estimates (e.g. regression coefficient) AND variation (e.g. standard deviation) or associated estimates of uncertainty (e.g. confidence intervals)
☐	☒	For null hypothesis testing, the test statistic (e.g. *F*, *t*, *r*) with confidence intervals, effect sizes, degrees of freedom and *P* value noted<br>*Give P values as exact values whenever suitable.*
☒	☐	For Bayesian analysis, information on the choice of priors and Markov chain Monte Carlo settings
☒	☐	For hierarchical and complex designs, identification of the appropriate level for tests and full reporting of outcomes
☒	☐	Estimates of effect sizes (e.g. Cohen's *d*, Pearson's *r*), indicating how they were calculated

Our web collection on statistics for biologists contains articles on many of the points above.

Software and code

Policy information about availability of computer code

Data collection	We conducted RNA sequencing (RNA-Seq) on total RNA from the FN3K KO and the wild-type (WT) cell lines. RNA extraction, preparation of RNA library and transcriptome sequencing were conducted by Novogene Co., LTD.
Data analysis	Human Protein Atlas (HPA) Version: The mRNA expression levels data was obtained using HPA version 23.0.<br>Ensembl Version: The mRNA data was also based on Ensembl version 109.<br>Xcalibur Software: The mass spectrometry data analysis was conducted using Xcalibur software version 2.2.<br>Thermo Proteome Discoverer: Used for protein identification and modification characterization, operating on version 1.4.<br>Mascot: Used within Proteome Discoverer for searching the database, version 2.7.<br>STRING Database Version: Used for pathway enrichment analysis, specifically version 11.5 mentioned for gene context.<br>Cytoscape 3.10.1: Network analysis<br>HISAT2: TMap trimmed sequence reads to the GRCh38.p5<br>StringTie v2.0.6: Transcript assembly<br>Ballgown v2.26: Differentially Expressed Genes analysis

For manuscripts utilizing custom algorithms or software that are central to the research but not yet described in published literature, software must be made available to editors and reviewers. We strongly encourage code deposition in a community repository (e.g. GitHub). See the Nature Portfolio guidelines for submitting code & software for further information.

Data

Policy information about availability of data

All manuscripts must include a data availability statement. This statement should provide the following information, where applicable:

- Accession codes, unique identifiers, or web links for publicly available datasets
- A description of any restrictions on data availability
- For clinical datasets or third party data, please ensure that the statement adheres to our policy

All relevant data supporting the findings of this study are available within the article (and its supplementary information files). RNA expression data for the WT and the FN3K KO samples have been deposited in the Gene Expression Omnibus database under the accession code GSE242555.

Research involving human participants, their data, or biological material

Policy information about studies with human participants or human data. See also policy information about sex, gender (identity/presentation), and sexual orientation and race, ethnicity and racism.

Reporting on sex and gender	N/A
Reporting on race, ethnicity, or other socially relevant groupings	N/A
Population characteristics	N/A
Recruitment	N/A
Ethics oversight	N/A

Note that full information on the approval of the study protocol must also be provided in the manuscript.

Field-specific reporting

Please select the one below that is the best fit for your research. If you are not sure, read the appropriate sections before making your selection.

☒ Life sciences ☐ Behavioural & social sciences ☐ Ecological, evolutionary & environmental sciences

For a reference copy of the document with all sections, see nature.com/documents/nr-reporting-summary-flat.pdf

Life sciences study design

All studies must disclose on these points even when the disclosure is negative.

Sample size	A sample size calculation was not conducted. For RNA sequencing and biochemical assays, a minimum of three replicates were used to enable the calculation of standard deviation and to maintain a manageable experimental sample size.
Data exclusions	N/A
Replication	Experiments conducted with three or more biological replicates.
Randomization	N/A
Blinding	N/A

Reporting for specific materials, systems and methods

We require information from authors about some types of materials, experimental systems and methods used in many studies. Here, indicate whether each material, system or method listed is relevant to your study. If you are not sure if a list item applies to your research, read the appropriate section before selecting a response.

Materials & experimental systems

n/a	Involved in the study
☐	☒ Antibodies
☐	☒ Eukaryotic cell lines
☒	☐ Palaeontology and archaeology
☒	☐ Animals and other organisms
☒	☐ Clinical data
☒	☐ Dual use research of concern
☒	☐ Plants

Methods

n/a	Involved in the study
☒	☐ ChIP-seq
☒	☐ Flow cytometry
☒	☐ MRI-based neuroimaging

Antibodies

Antibodies used

FN3K Polyclonal Antibody - Source: Invitrogen, Catalog #: PA5-28603
FN3K for Immunofluorescence - Source: Invitrogen, Catalog #: PA5-66239
Tomm20 - Source: Abnova, Catalog #: H00009804-M01
Fatty Acid Synthase (FASN) - Source: Invitrogen, Catalog #: MA-531490
Lactate Dehydrogenase A (LDHA) - Source: Cell Signaling Technology, Catalog #: C4B5
Vinculin - Source: Cell Signaling Technology, Catalog #: E1E9V
Histone H3 - Source: Cell Signaling Technology, Catalog #: D1H2
Succinate Dehydrogenase Complex Subunit A (SDHA) - Source: Cell Signaling Technology, Catalog #: 5839S
His Tag - Source: Cell Signaling Technology, Catalog #: 27E8

Validation

Eukaryotic cell lines

Policy information about cell lines and Sex and Gender in Research

Cell line source(s)

HepG2 cells, HB-8065(bought from American Type Culture Collection (ATCC))

Authentication

FN3K KO was authenticated using western blot

Mycoplasma contamination

Reported as not detected

Commonly misidentified lines
(See ICLAC register)

Name any commonly misidentified cell lines used in the study and provide a rationale for their use.

Plants

Seed stocks

N/A

Novel plant genotypes

N/A

Authentication

N/A
